# Structure-guided design of a PCSK9 epitope vaccine with efficacy against hyperlipidemia and atherosclerosis

**DOI:** 10.1093/lifemeta/loag013

**Published:** 2026-05-26

**Authors:** Hongliang Sun, Zhuang Li, Xinli Hu, Xuemei Zhang, Kun Ma, Jian Zhang, Chang Liu, Ruiping Xiao

**Affiliations:** College of Future Technology, Peking University, Beijing 100871, China; Vaccine Division I, Changchun Institute of Biological Products Co., Ltd, Changchun, Jilin 130012, China; First Research Laboratory, Changchun Institute of Biological Products Co., Ltd, Changchun, Jilin 130012, China; College of Future Technology, Peking University, Beijing 100871, China; Department of Scientific Research Management, Peking Institute of Biological Products Co., Ltd, Beijing 100176, China; Vaccine Division I, Changchun Institute of Biological Products Co., Ltd, Changchun, Jilin 130012, China; Laboratory Animal Department, Changchun Institute of Biological Products Co., Ltd, Changchun, Jilin 130012, China; Vaccine Division I, Changchun Institute of Biological Products Co., Ltd, Changchun, Jilin 130012, China; College of Future Technology, Peking University, Beijing 100871, China

**Keywords:** PCSK9, Alphafold3, hyperlipidemia, atherosclerosis, vaccine

## Abstract

Proprotein convertase subtilisin/kexin type 9 (PCSK9) plays a central role in regulating low-density lipoprotein cholesterol (LDL-C) levels and has emerged as an attractive target for atherosclerotic cardiovascular disease (ASCVD) therapy. While mono­clonal antibodies targeting PCSK9 have demonstrated clinical efficacy, their high cost and need for repeated administration limit widespread use. In this study, we developed a peptide-based vaccine by identifying B-cell epitopes from PCSK9–antibody complexes using the Protein Data Bank (PDB) structural data and AlphaFold3 prediction, and fusing them with a heterologous T-helper epitope. The vaccine induced strong and durable anti-PCSK9 antibody responses in mice, guinea pigs, and rhesus macaques when formulated with the CpG plus alum adjuvant. The vaccine significantly reduced LDL-C levels and attenuated hepatic lipid accumulation in both prophylactic and therapeutic mouse models. Moreover, it mitigated the progression of atherosclerotic plaques. The vaccine also demonstrated no signs of systemic toxicity or autoimmunity in animal models. These findings indicate that the vaccine is a safe, effective, and scalable approach for controlling hypercholesterolemia and preventing ASCVD through active immunization against PCSK9.

## Introduction

Cardiovascular diseases (CVDs) remain the leading cause of morbidity and mortality worldwide, with atherosclerosis serving as the primary pathological basis in most cases. Among the major risk factors for atherosclerosis, hyperlipidemia, particularly elevated low-density lipoprotein cholesterol (LDL-C) levels, plays a central role [1, 2]. According to the World Health Organization (WHO), more than 39% of adults aged 25 years and older globally have elevated cholesterol levels, contributing to over 4.5 million deaths each year [3]. In China, CVDs have consistently ranked as the top cause of mortality, accounting for approximately two out of every five deaths. With rapid urbanization and lifestyle changes, the burden of dyslipidemia has risen sharply, with recent national surveys indicating that nearly 40% of adults exhibit abnormal lipid profiles [4]. As such, effective control of plasma cholesterol levels, especially LDL-C, has become a critical strategy for the prevention and management of atherosclerotic cardiovascular disease (ASCVD).

A central regulator of LDL-C metabolism is the proprotein convertase subtilisin/kexin type 9 (PCSK9), a serine protease predomi­nantly secreted by the liver [5, 6]. The catalytic domain of PCSK9 interacts with LDL-C receptors (LDLRs) located on the surface of hepatocytes, triggering their internalization and subsequent degradation within lysosomes. Gain-of-function mutations in PCSK9 lead to premature atherosclerosis, whereas loss-of-­function mutations are associated with reduced LDL-C levels and cardiovascular risk [7, 8]. The pivotal role of the PCSK9−LDLR axis in lipid regulation has made it an attractive target for therapeutic intervention [9].

Currently, several therapeutic strategies have been developed to manage clinical dyslipidemia. These include statins, monoclonal antibodies such as evolocumab, alirocumab, and tafolecimab, and small interfering RNAs (siRNAs) such as inclisiran. Statins, which reduce endogenous cholesterol synthesis through inhibiting 3-hydroxy-3-methylglutaryl-coenzyme A (HMG-CoA) reductase and promoting LDL-C clearance, remain the standard first-line therapy [10]. However, their use is often associated with side effects such as muscle-related symptoms and hepatotoxicity, contributing to poor patient compliance and treatment discontinuation [11, 12]. Monoclonal antibodies targeting PCSK9 prevent its interaction with LDLRs, thereby enhancing LDL-C clearance [13–15]. In combination with statins, these antibodies provide an incremental LDL-C reduction of 50%–60% beyond that achieved with statin therapy alone [16, 17]. Nonetheless, such therapies require regular subcutaneous injections, typically every 2–4 weeks, which may limit long-term compliance [18]. SiRNA-based approaches, such as inclisiran, act by silencing hepatic PCSK9 expression [19, 20]. This modality allows for infrequent dosing—initial administration followed by a second dose at 3 months and maintenance injections every 6 months thereafter. Oral small-molecule inhibitor CVI-LM001 blocks the endocytosis and degradation of LDLR, thereby reducing plasma LDL-C levels; however, it remains in the clinical stage. Despite their proven efficacy, the widespread adoption of PCSK9-targeted inhibitors remains hindered by high costs and the need for repeated administration [21]. Thus, there is an urgent need to develop alternative strategies that offer both robust lipid-lowering effects and improved accessibility, affordability, and convenience for long-term management and prevention of ASCVD.

Vaccines have long played a critical role in preventing infectious diseases and are now being investigated for the prevention and treatment of chronic diseases [22]. In this context, a PCSK9-targeted vaccine offers the potential for a long-term, cost-effective strategy to control LDL-C levels and reduce cardiovascular risk. Previous PCSK9 vaccine designs have mainly employed the entire catalytic domain of PCSK9 as the immunogen or selected epitopes based on the structural interface between PCSK9 and LDLR [23]. In contrast, the present study adopted a distinct epitope-design strategy. Epitope regions were identified by integrating structural information from AlphaFold3-predicted PCSK9 and experimentally determined structures available in the Protein Data Bank (PDB). This antibody-structure-guided approach provides a targeted rationale for epitope selection and represents a key innovative aspect of this study. To enhance immunogenicity and minimize the risk of immune tolerance, heterologous viral T-helper (Th) epitopes were incorporated into the vaccine construct. We evaluated the efficacy of the vaccine in multiple mouse models and non-human primates (NHPs), aiming to assess its potential for both prophylactic and therapeutic use in hypercholesterolemia. Our results suggest that a PCSK9 epitope-based vaccine, combined with Th epitope, is a promising therapeutic modality for the prevention and treatment of hyperlipidemia and atherosclerosis.

## Results

### Epitope selection based on structural and conservation analysis

**Figure 1:**
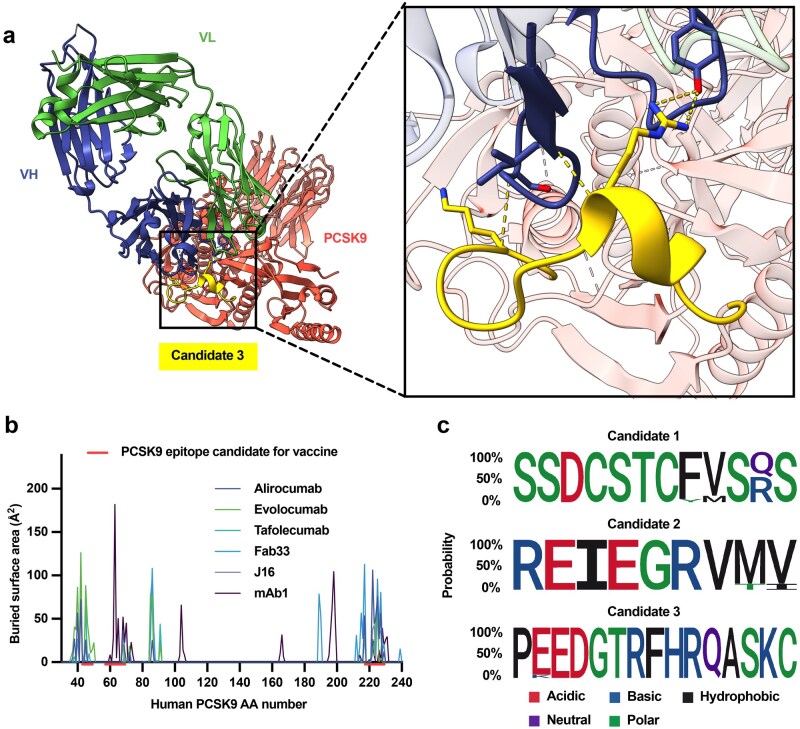
Identification of epitope candidates from PCSK9−antibody complexes. (a) The interaction between PCSK9 and the corresponding antibody from PDB repository (PDB: 3H42). The PCSK9 protein is shown in blue, and the heavy and light chains of the antibody are shown in green and purple, respectively. The epitope candidate 3 is in yellow. (b) Analysis of BSA at the PCSK9-antibody interface. Regions with higher values (red) indicate potential epitope candidates. The human PCSK9 amino acid numbers were assigned starting from the first amino acid of the PCSK9 catalytic domain. AA, amino acid. (c) The analysis of amino acid conservation of epitope candidates. See also Supplementary Figs. S1 and S2.

Using AlphaFold3 predictions and available crystal structures in the PDB, we obtained six PCSK9−antibody complex structures in total (Fig. 1a; Supplementary Fig. S1). For each structure, we calculated the buried surface area (BSA) of individual PCSK9 residues at the antibody-binding interface. Based on these values, we selected three peptide regions with the highest BSA as candidate epitopes (Fig. 1b). As an example, candidate peptide 3 exhibited a total BSA of 466.7 Å^2^ when bound to the antibody and formed multiple stable hydrogen bonds across the interface. These features suggest that Peptide 3 possesses a high potential to serve as a conformational B-cell epitope and may play a critical role in antigen recognition.

To further validate their potential, we performed multiple sequence alignment (MSA) of PCSK9 orthologs from 19 different species. All three candidate peptides exhibited high sequence conservation across all examined species (Fig. 1c; Supplementary Fig. S2). This high level of interspecies conservation implies two key points: (i) these peptides are likely located within functionally essential domains of PCSK9 and (ii) immune responses elicited by these epitopes in animal models, especially rodents and NHPs, are expected to be translatable to human settings. Collectively, these data support the use of these three peptides as promising antigenic candidates for vaccine development.

### The PVC3 vaccine induces robust antibody responses in mice

**Figure 2:**
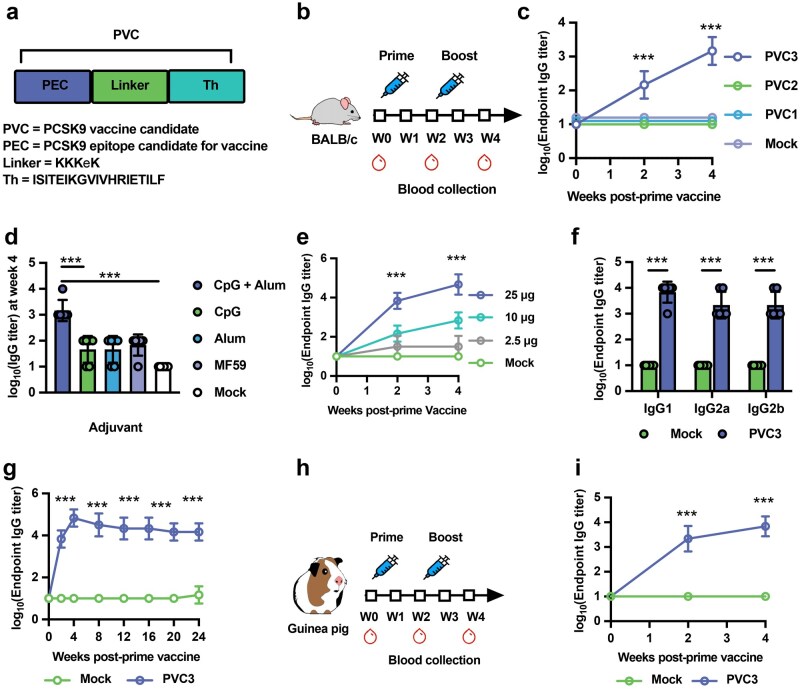
Immunogenicity of the PCSK9 vaccine in mice and guinea pigs. (a) Schematic representation of the vaccine construct, showing the PCSK9 candidate epitope linked to a Th epitope via a flexible linker. (b) Timeline of the immunization protocol in BALB/c mice, with primary immunization at week 0 and boost at week 2. Each (10 μg) of the three candidate vaccines was mixed with the CpG + Alum composite adjuvant. Physiological saline was used as the control, 100 μL of which was injected subcutaneously into BALB/c mice. (c) Comparison of antibody responses induced by different PVCs. Blood samples were collected at 0, 2, and 4 weeks, and sera were separated. Anti-PCSK9 antibodies were detected using the ELISA method, with results presented as base-10 logarithms. (d) Comparison of antibody responses induced by PCV3 vaccine with different adjuvant formulations in BALB/c mice. CpG + Alum, combination of CpG and aluminum adjuvant; CpG, CpG adjuvant alone; Alum, aluminum adjuvant alone; MF59: MF59 adjuvant; Mock: blank control group. The mouse immunization, blood collection, and antibody detection methods are consistent with those in (b). (e) Dose-dependent antibody response in mice immunized with 2.5, 10, or 25 μg PVC3 vaccine. The mouse immunization, blood collection, and antibody detection methods are consistent with those in (b). (f) Isotype distribution (IgG1, IgG2a, and IgG2b) of anti-PCSK9 antibodies in mice immunized with the 25 μg dose. Serum was collected at week 4 and analyzed by ELISA method. (g) Persistence of antibody response in mice over a 24-week period. (h) Timeline of the immunization protocol in guinea pigs, with primary immunization at week 0 and boost at week 2. Blood samples were collected and serum was separated at 0, 2, and 4 weeks. (i) Time-course curve showing PCSK9-specific IgG titers in sera from guinea pigs. All data are presented as mean ± standard error of the mean (SEM). The sample size was *n *= 6 per group. Unpaired Student’s *t*-test (f), one-way ANOVA with Tukey’s *post hoc* test (d), and two-way ANOVA with Sidak’s *post hoc* test (a, e, g, and i) were used for statistical analysis. *P < 0.05; **P < 0.01; ***P < 0.001.

The selected PCSK9 epitope candidates are short linear peptides of approximately 10 amino acids in length. Due to their limited size, these minimal epitopes typically exhibit poor immunogenicity when administered alone [24]. To address this, a Th epitope was appended to the C-terminus of the PCSK9 epitope via a fle­xible linker. Each PCSK9 vaccine candidate (PVC) thus adopted a common modular structure of “PCSK9 epitope−KKKeK−Th epitope” (Fig. 2a).

To evaluate the immunogenicity of candidate vaccines, BALB/c mice were subcutaneously immunized with 10 μg doses of each vaccine formulated with a CpG plus aluminum hydroxide (CpG + Alum) adjuvant (Fig. 2b). Antibody responses specific to PCSK9 were assessed by enzyme-linked immunosorbent assay (ELISA). Among the three candidates, only PVC3 elicited a robust IgG response against PCSK9. The IgG titers progressively increased and reached a geometric mean titer (GMT) of approximately 3.2 (log_10_) by week 4 (Fig. 2c). In contrast, PVC1 and PVC2 failed to induce any detectable PCSK9-specific antibodies above baseline levels throughout the observation period, indicating their lack of immunogenicity.

To identify the optimal adjuvant for the PVC3 peptide vaccine, we tested four formulations: Alum alone (a conventional Th2-biased adjuvant), CpG oligodeoxynucleotide alone (a potent Toll-like receptor 9 [TLR9] agonist that promotes Th1-biased responses), MF59 (a squalene-based oil-in-water emulsion approved for human influenza vaccines), and a combination of Alum and CpG (a mixed Th1/Th2 adjuvant) [25–27]. Sera collected at day 28 showed that the Alum + CpG formulation significantly outperformed the others, inducing the highest titers of PCSK9-specific antibodies (*P *< 0.001 versus all other groups; Fig. 2d). While both Alum and CpG alone generated detectable antibody responses, titers were substantially lower than those observed with the combined formulation. Interestingly, despite its efficacy in other vaccine platforms [28], MF59 did not elicit a superior response in this peptide-based PCSK9 vaccine model. Based on these results, the Alum + CpG adjuvant combination was selected as the standard formulation for all subsequent studies.

To determine the optimal immunization dose, BALB/c mice were vaccinated with 2.5 μg, 10 μg, or 25 μg of PVC3 formulated with Alum + CpG. The 25 μg dose induced a significantly higher antibody titer (GMT 4.7) compared to the 10 μg group (GMT 2.8), and the difference was statistically significant (*P *< 0.001; Fig. 2e).

To further characterize the immune response elicited by PVC3, we evaluated IgG subclasses in serum samples collected at week 4. PVC3 vaccination induced robust titers of PCSK9-specific IgG1, IgG2a, and IgG2b, all above GMT of 3 (Fig. 2f). IgG1 was the domi­nant subclass, with a GMT of 3.8, significantly higher than that in control animals (*P *< 0.001), suggesting a strong Th2-skewed immune response. The presence of multiple IgG subclasses indicates a broad and balanced humoral response, which may contribute to more comprehensive immunoprotection.

The durability of the antibody response was assessed by mea­suring serum titers up to week 24. By that time, the antibody GMT remained at 4.2, with no significant decline observed compared to earlier time points, indicating that PVC3-induced humoral immunity is long-lasting (Fig. 2g).

To evaluate the cross-species immunogenicity of PVC3, guinea pigs were immunized with a 50 μg dose of the vaccine (Fig. 2h). By week 4, PCSK9-specific IgG titers reached a GMT of 3.8, confirming strong immunogenicity in this species as well (Fig. 2i).

Together, these findings indicate that the PVC3 vaccine elicits a potent, sustained antibody response against PCSK9, supporting its potential as an effective vaccine.

### The PCSK9 vaccine demonstrates a favorable safety profile

**Figure 3:**
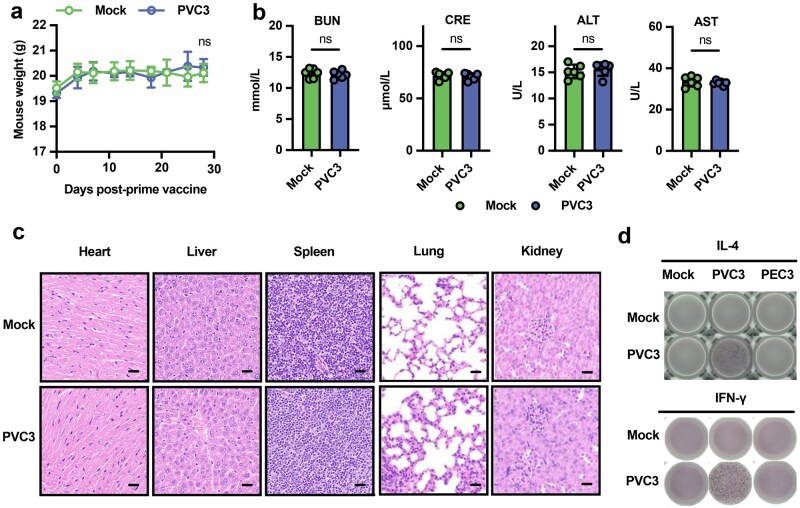
Safety assessment of the PCSK9 vaccine. (a) Body weight monitoring of BALB/c mice immunized with the vaccine or adjuvant control over 4 weeks. (b) Serum biochemical marker analyses. Following inoculation of BALB/c mice with 25 μg PVC3 vaccine and mock control, blood was collected at week 12 for serum separation. Serum biochemical indicators of liver and kidney function were then detected using appropriate assay kits (BUN, CRE, ALT, and AST). (c) Representative H&E staining of major organs (the heart, liver, spleen, lungs, and kidneys) from immunized and control mice at the end-point of the experiment. Scale bars: 20 μm. (d) ELISPOT analysis of IFN-γ and IL-4 production by splenocytes in response to PVC3 vaccine, PEC3, or mock. Mice were euthanized, and the spleens were harvested at week 4. All data are presented as mean ± SEM. The sample size was *n *= 6 per group. Unpaired Student’s *t*-test (b) and Two-way ANOVA with Sidak’s *post hoc* test (a) were used for statistical analysis. *P < 0.05; **P < 0.01; ***P < 0.001.

Vaccine safety is essential to ensure clinical feasibility and long-term reliability. In this study, no significant differences in body weight were observed between vaccinated and control mice, indicating no overt systemic toxicity (Fig. 3a). Serum biochemical markers, including blood urea nitrogen (BUN), alanine aminotransferase (ALT), creatinine (CRE), and aspartate aminotransferase (AST), remained within physiological ranges across all groups, suggesting no evidence of kidney or liver dysfunction (Fig. 3b). Histopathological analysis of major organs (the heart, liver, spleen, lungs, and kidneys) revealed no structural abnormalities or inflammatory cell infiltration in vaccinated animals (Fig. 3c), further supporting the favorable safety profile of the vaccine.

To evaluate the potential risk of autoimmune responses, we performed the enzyme-linked immunospot/immunosorbent spot (ELISPOT) assays to detect cytokine production by splenocytes following immunization. Robust interferon-γ (IFN-γ) and interleukin-4 (IL-4) responses were observed in splenocytes stimulated with the full PVC3 vaccine, confirming T cell activation (Fig. 3d). In contrast, stimulation with the PCSK9 B-cell epitope (PEC3) alone failed to elicit detectable cytokine secretion, indicating that the epitope by itself did not trigger T cell-mediated immunity. This suggests that the immune system does not recognize endogenous PCSK9 as a self-antigen, thereby minimizing the risk of autoimmunity.

### Preventive effect of the PCSK9 vaccine in mice

**Figure 4:**
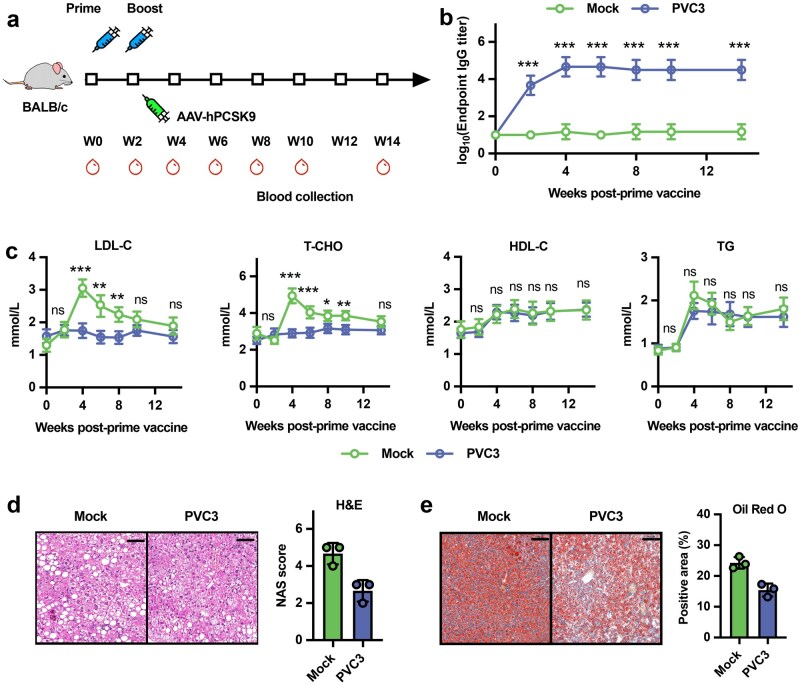
Efficacy of the PCSK9 vaccine in a preventive mouse model. (a) Experimental design for the preventive model. BALB/c mice were subcutaneously immunized twice. At week 2, the immunized mice received tail-vein injections of AAV-hPCSK9^D374Y^. (b) Anti-PCSK9 antibody titers over time. Mouse blood samples were collected every 2 weeks, and antibodies were detected by using ELISA (0–14). (c) Blood lipid profiles in mice measured using a lipid detection kit. LDL-C (left), T-CHO (middle left), HDL-C (middle right), and TG (right). (d) Representative H&E staining (left) and quantitative analyses (right) of liver tissues from immunized and control mice at the end-point of the experiment. (e) Representative Oil Red O staining (left) and quantitative analyses (right) of liver tissues from immunized and control mice at the end-point of the experiment. All data are presented as mean ± SEM. Two-way ANOVA with Sidak’s *post hoc* test (b and c) was used for statistical analysis. *P < 0.05; **P < 0.01; ***P < 0.001. Scale bars: 100 μm.

To investigate the preventive efficacy of the PVC3 vaccine against hypercholesterolemia, we employed a mouse model induced by intravenous administration of adeno-associated virus encoding human PCSK9^D374Y^ (AAV-hPCSK9^D374Y^). BALB/c mice were subcutaneously immunized with the PVC3 vaccine at weeks 0 and 2, followed by tail-vein injection of AAV at week 2 (Fig. 4a). Consistent with previous results, the vaccine elicited a robust PCSK9-specific antibody response, with GMT exceeding 4.6 (*P *< 0.001) and sustained for 14 weeks (Fig. 4b).

At week 2, prior to AAV administration, the mock and PVC3-vaccinated groups exhibited comparable baseline LDL-C levels (1.79 versus 1.76 mmol/L; *P *> 0.99). By week 4, following AAV administration, serum LDL-C levels markedly increased in the mock group, whereas no such elevation was observed in PVC3-vaccinated mice (3.05 versus 1.75 mmol/L; *P *< 0.05), indicating a significant inhibitory effect of the vaccine on LDL-C elevation (Fig. 4c, left panel). Total cholesterol (T-CHO) levels showed the same pattern at week 4, with a clear inhibitory effect observed in the vaccinated group (4.94 versus 2.90 mmol/L; *P *< 0.05) (Fig. 4c, middle left). Notably, no differences were observed for the levels of high-density lipoprotein cholesterol (HDL-C) and triglycerides (TG) across groups, suggesting the selectivity of the vaccine toward LDL-C modulation (Fig. 4c, middle right and right). Histological analysis further supported these findings: both hematoxylin and eosin (H&E) staining and Oil Red O staining revealed reduced hepatic lipid accumulation in PVC3-treated mice compared to the controls (Fig. 4d and e), indicating a protective effect of the vaccine against hepatic steatosis.

### Therapeutic effect of the PCSK9 vaccine in mice

**Figure 5:**
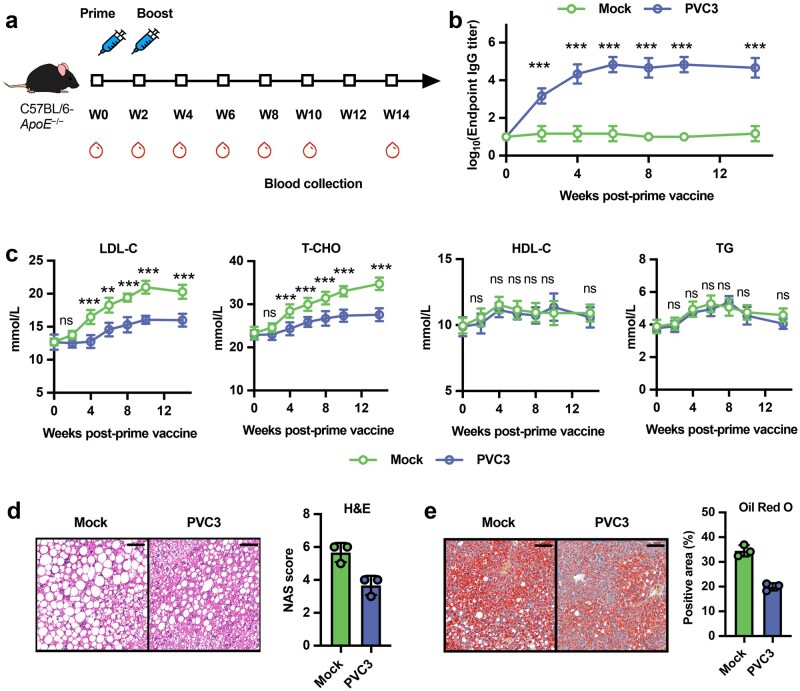
Efficacy of the PCSK9 vaccine in a therapeutic mouse model. (a) Experimental design for the therapeutic *ApoE*^−/−^ model. *ApoE*^−/−^ mice were subcutaneously immunized twice at weeks 0 and 2. Blood samples were collected every 2 weeks from week 0 through week 14. (b) Anti-PCSK9 antibody titers over time. Following the separation of serum from blood samples collected between weeks 0 and 14, antibodies were detected using the ELISA method. (c) Blood lipid profiles in mice measured using the lipid detection kit. LDL-C (left), T-CHO (middle left), HDL-C (middle right), and TG (right). (d) Representative H&E staining (left) and quantitative analyses (right) of liver tissues from immunized and control mice at the end-point of the experiment. (e) Representative Oil Red O staining (left) and quantitative analyses (right) of liver tissues from immunized and control mice at the end-point of the experiment. All data are presented as mean ± SEM. Two-way ANOVA with Sidak’s *post hoc* test (b and c) was used for statistical analysis. *P < 0.05; **P < 0.01; ***P < 0.001. Scale bars: 100 μm.

To assess the therapeutic potential of the PVC3 vaccine in the model of dyslipidemia, we utilized *ApoE*^−/−^ mice, which spontaneously develop hypercholesterolemia and atherosclerotic lesions. Mice (17–18 weeks old) were subcutaneously immunized with two doses of PVC3 vaccine at weeks 0 and 2. Serum samples were collected biweekly up to week 14 (Fig. 5a). Vaccinated mice deve­loped robust PCSK9-specific IgG responses, with GMT reaching 4.7 by week 4 (Fig. 5b), indicating successful induction of a humoral response.

Serum lipid analysis revealed that PVC3 vaccination significantly attenuated the elevation of LDL-C levels compared to the mock group. In control mice, LDL-C levels remained persistently high throughout the observation period, reaching 16.5 mmol/L at week 4 and 20.3 mmol/L at week 14. In contrast, PVC3-vaccinated mice exhibited a markedly slower rate of increase, with LDL-C levels of 12.7 mmol/L at week 4 and 16.0 mmol/L at week 14, corresponding to reductions of 29% and 20%, respectively, relative to the controls (*P *< 0.01) (Fig. 5c, left). Similar reductions were observed in T-CHO levels (Fig. 5c, middle left), while HDL-C and TG levels remained unaffected (Fig. 5c, middle right and right), mirroring the lipid profile seen in the preventive model.

Histopathological evaluation of liver tissues demonstrated reduced lipid accumulation in the hepatocytes of vaccinated mice, as confirmed by H&E and Oil Red O staining (Fig. 5d and e). These findings indicate that the PVC3 vaccine not only prevents but also therapeutically mitigates hyperlipidemia-associated pathology in mice.

### The PCSK9 vaccine attenuates atherosclerotic lesion development

**Figure 6:**
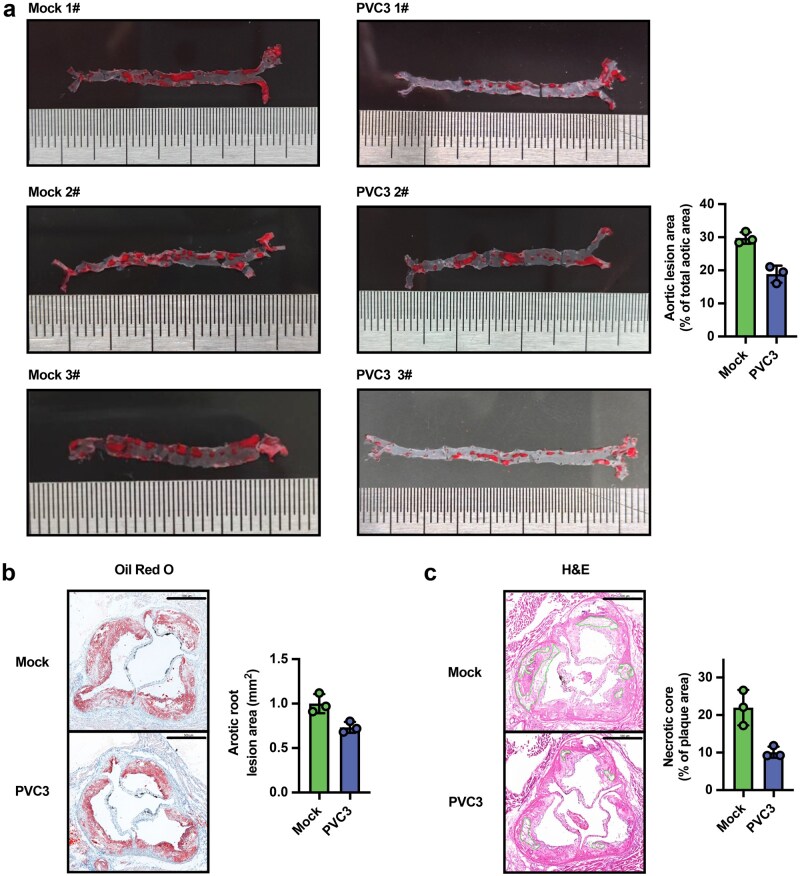
Effect of the PCSK9 vaccine on atherosclerosis. (a) Representative images of Oil Red O-stained aortas from vaccinated and control *ApoE*^−/−^ mice at the end of the therapeutic study (week 14). (b) Measurement of the aortic root atherosclerotic lesion area at week 14. (c) Representative H&E staining of aortic root sections from vaccinated and control mice at week 14, with quantification of necrotic core area. Scale bars: 500 μm.

Given the potent lipid-lowering effect of the PVC3 vaccine, we next assessed its impact on the progression of atherosclerosis in *ApoE^−/−^* mice. Oil Red O staining of the whole aorta revealed that mice receiving the vaccine exhibited a significantly reduced aortic lesion area (18.9% of total aortic surface) compared to the controls (29.8%), indicating a substantial reduction in overall atherosclerotic burden (Fig. 6a).

In addition, cross-sectional analysis of the aortic root demonstrated that PVC3 vaccination markedly decreased lesion area (0.7 mm^2^; Fig. 6b) compared with the mock group (1.0 mm^2^), and reduced the proportion of necrotic core relative to total plaque area (10.1%) compared with the mock group (22.0%; Fig. 6c). These findings suggest that the vaccine not only lowers circulating LDL-C levels but also confers direct protection against atherosclerotic lesion formation and progression.

### The PCSK9 vaccine induces antibody responses in NHPs

**Figure 7:**
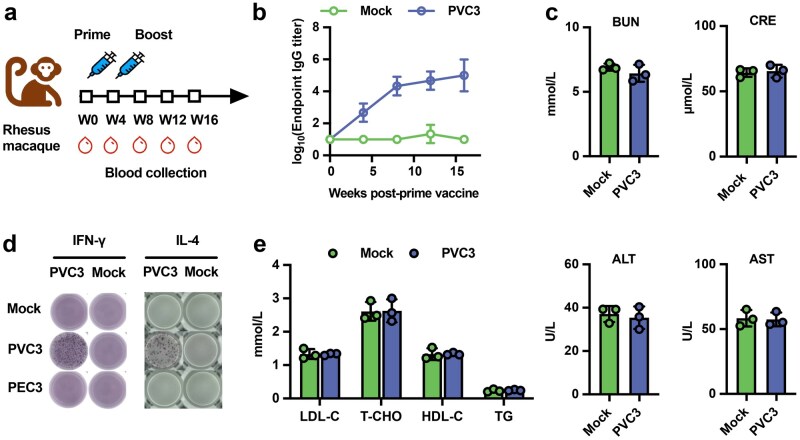
Evaluation of the PCSK9 vaccine in NHPs. (a) Timeline of the immunization protocol in rhesus macaques, with immunizations at weeks 0 and 4. Blood samples were collected at 4-week intervals, and sera were separated for ELISA detection of anti-PCSK9 antibodies. (b) Anti-PCSK9 antibody titers in vaccinated and control monkeys over time (*n *= 3 per group). (c) Serum biochemical parameters (BUN, CRE, ALT, and AST) in vaccinated and control monkeys. The assay kits were used for detecting sera at week 16. (d) ELISPOT analyses of IFN-γ and IL-4 production by peripheral blood mononuclear cells (PBMCs) from vaccinated and control monkeys at week 8 in response to PVC3 vaccine, PEC3, or mock. (e) Serum lipid parameters (LDL-C, T-CHO, HDL-C, and TG) in vaccinated and control monkeys detected at week 16. All data are presented as mean ± SEM.

To further assess the translational potential of the PVC3 vaccine, we conducted studies in rhesus macaques using a two-dose intramuscular immunization regimen (Fig. 7a). The vaccine elicited a robust PCSK9-specific antibody response, with GMT exceeding 4.3 at week 8 (*P *< 0.001) and sustained for 16 weeks (Fig. 7b). Serum biochemical parameters, including BUN, ALT, CRE, and AST, remained within normal physiological ranges across all groups, indicating no signs of hepatic or renal toxicity (Fig. 7c). Moreover, ELISPOT analysis revealed no evidence of aberrant IFN-γ or IL-4 responses against endogenous PCSK9 epitopes, further confirming the absence of autoimmune activation in NHPs (Fig. 7d).

Vaccinated monkeys developed robust anti-PCSK9 IgG responses, with titers similar to those observed in mice and guinea pigs (Fig. 2b and h). However, no statistically significant differences were observed in plasma lipid parameters, including LDL-C, TC, HDL-C, and TG, compared to the control group (*P *> 0.05 for all; Fig. 7e). These results suggest that while the PVC3 vaccine elicits robust immunogenicity in rhesus macaques, its lipid-lowering efficacy may vary in larger animal models and warrants further optimization.

## Discussion

In recent years, PCSK9 has emerged as a major therapeutic target for the management of hypercholesterolemia and ASCVD, attracting substantial research interest. Currently, PCSK9 inhibitors face several challenges, including short dosing intervals for monoclonal antibodies that result in poor patient compliance, high costs associated with siRNA therapies, and small-molecule candidates remaining in the clinical trial phase. PCSK9 vaccine development holds promise for enhancing therapeutic efficacy, improving patient compliance, and enabling long-term lipid management. Several PCSK9 vaccine strategies have been reported, including PCSK9 mimic [23], nanoparticle [29, 30], virus-like particle [31–33], and toxin-conjugated constructs [34–36]. In this study, we employed a simple and distinct design by developing a peptide-based epitope vaccine. Immunogenic regions were identified through structural analysis of PCSK9–antibody complexes, and the selected B-cell epitopes were fused with a heterologous Th epitope to enhance immunogenicity. Our findings demonstrate that this vaccine successfully induced antibodies and reduced circulating LDL-C levels in mouse models. These results highlight the potential of epitope-based PCSK9 vaccines as a promising stra­tegy for cholesterol control and ASCVD prevention.

To enhance their immunogenic potential, each PCSK9 epitope was fused to a measles virus-derived Th epitope [37, 38]. This Thepitope is well-known for its potent immune adjuvant effect and cross-species conservation, as it can effectively activate CD4^+^ T cells and promote humoral immune responses. To ensure appropriate spatial separation and flexibility between the PCSK9 epitope and the Th epitope, a short flexible linker consisting of four lysine resi­dues (KKKK) was inserted between the two domains. This stra­tegy serves two key purposes. First, Th epitopes can specifically activate CD4^+^ Th cells, thereby amplifying the humoral immune response directed against the PCSK9 epitope. Second, incorporation of a heterologous Th sequence introduces molecular heterogeneity to the peptide vaccine construct, effectively reducing the risk of immune tolerance that may arise from repeated exposure to self-antigens. Importantly, this strategy likely contributes to the excellent safety profile of the vaccine. Throughout the course of the study, we observed no signs of systemic toxicity, hepatic injury, or renal impairment in any vaccinated animals. Moreover, ELISPOT assays confirmed the absence of harmful autoimmune responses. These results help alleviate concerns regarding the risk of autoimmune activation or immune tolerance associated with vaccines targeting self-proteins such as PCSK9.

Among the three candidate peptides, only PVC3 elicited a detectable and robust antibody response, whereas PVC1 and PVC2 failed to induce significant immunogenicity *in vivo*. This discrepancy prompted further investigation into possible structural and functional determinants. Notably, although PVC1 and PVC2 exhibited relatively high BSA values in most of the PCSK9–antibody complexes analyzed (Supplementary Fig. S1), their inability to trigger an immune response suggests that surface exposure alone is insufficient to predict immunogenicity. Factors such as epitope flexibility, conformational dynamics, and accessibility in the native protein context likely play critical roles in shaping effective B-cell recognition [39, 40]. Interestingly, structural ana­lysis revealed that the elevated BSA value of PVC3 was observed exclusively in the crystal structure of the PCSK9–antibody complex (PDB: 3H42) [41], but not in the other two complexes (PDB: 5VL7 and 3SQO) [42, 43]. Closer inspection of the latter structures showed that the peptide region corresponding to PVC3 was either unresolved or partially missing, indicating a high degree of structural flexibility or intrinsic disorder in this region. The lack of defined electron density implies that PVC3 resides in a dynamic or conformationally mobile domain of PCSK9, potentially representing a functionally important epitope recognized by neutralizing antibodies. This structural plasticity also explains why the PVC3 epitope was not identified by AlphaFold3, which primarily models stable structural elements with high confidence [44]. Collectively, these observations suggest that PVC3 corresponds to a conformationally flexible yet functionally critical binding interface, which may contribute to its immunogenicity. These findings highlight the importance of incorporating structural dynamics and multi-template structural comparison into rational B-cell epitope selection for vaccine design.

Notably, despite the potent therapeutic and preventive efficacy of the PVC3 vaccine in lowering serum cholesterol levels and attenuating atherosclerotic plaque progression in mouse models, the present study revealed no statistically significant reductions in serum lipid indices in healthy rhesus macaques. Due to time and resource constraints, each experimental group included only three monkeys. However, we believe that the limited sample size is not the primary reason for this outcome. Interestingly, the induction of robust anti-PCSK9 antibody responses without concomitant reductions in blood lipid levels has also been documented in previous studies. For instance, a PCSK9 vaccination in NHPs elicited strong antibody responses but failed to lower serum lipid levels [45]. Similarly, an anti-PCSK9 peptide-based vaccine was ineffective at reducing LDL-C levels in both wild-type mice and Wistar rats, despite its clear immunogenicity [34]. Consistent with these observations, our preliminary data also showed that PVC3 vaccination did not significantly affect serum cholesterol in normal BALB/c mice fed a high-fat diet, even though high titers of anti-PCSK9 antibodies were generated. These findings prompted us to turn to more suitable hypercholesterolemic models, namely, *ApoE*^−/−^ mice and AAV-hPCSK9–injected mice, for the evaluation of lipid-lowering efficacy. Extending this rationale, assessing the performance of the vaccine in hypercholesterolemic NHP models would be even more appropriate. However, due to resource limitations, such models were not available in the present study. Another possible explanation for the lack of lipid reduction in healthy monkeys is that PCSK9 may not be the sole determinant of dyslipidemia [46]. The regulation of lipid metabolism is highly complex and involves multiple pathways, including hepatic lipid biosynthesis, lipoprotein remodeling and clearance, and inflammatory signaling. In normolipidemic organisms, compensatory mechanisms may offset the effects of PCSK9 inhibition [47]. Therefore, future research should consider combination strategies targeting complementary pathways or focus on stratifying patients with PCSK9-driven dyslipidemia to better realize the clinical potential of PCSK9-targeted vaccines [48].

In conclusion, we developed a novel peptide-based vaccine targeting PCSK9, guided by structural insights from PCSK9–antibody interactions. The vaccine developed in this study induced robust and durable antibody responses capable of reducing LDL-C levels and attenuating atherosclerosis in hypercholesterolemic mouse models. While the lack of lipid-lowering efficacy in NHPs presents a translational challenge, the insights gained from this comprehensive preclinical evaluation provide a foundation for further optimization and development of PCSK9-targeting vaccines for human applications.

## Limitations of the study

Given the lack of LDL-C lowering in the NHP model, the potential utility of this vaccine in combination with standard lipid-lowering agents such as statins remains to be evaluated. This study focused on the functional evaluation of the vaccine. Detailed molecular mechanisms were not assessed, including hepatic LDLR protein levels, circulating free PCSK9, and *in vitro* neutralization of PCSK9-mediated LDLR degradation. Future studies should aim to address these limitations to support the continued development and clinical translation of PCSK9-targeted vaccines.

## Materials and methods

### Epitope identification through PCSK9−antibody complexes

To identify potential B-cell epitopes of PCSK9 for vaccine design, two complementary strategies were employed to obtain PCSK9−antibody complex structures.

First, structural models of PCSK9 in complex with three clinically approved monoclonal antibodies, that is, evolocumab, alirocumab, and tafolecimab, were predicted using AlphaFold3 [49]. The antibody variable region sequences were retrieved from the IMGT database (the international ImMunoGeneTics information system), while the full-length human PCSK9 protein sequence (UniProt ID: Q8NBP7) was obtained from the UniProt database.

Second, we searched the PDB and identified three experimentally determined crystal structures of PCSK9−antibody complexes: PDB IDs 5VL7, 3SQO, and 3H42 [50].

For each complex, we analyzed the PCSK9−antibody interface using ChimeraX [51]. Candidate epitope regions were selected based on surface accessibility and high BSA values. In addition, interfacial hydrogen bond interactions were examined.

To evaluate the evolutionary conservation of candidate epitopes across species, multiple PCSK9 ortholog sequences were downloaded from the UniProt database [52]. MSA was performed using Clustal Omega [53], and sequence conservation was visualized using the ggseqlogo package in R [54].

### Vaccine design and preparation

With reference to the design strategy of VXX-401 vaccine [37], the selected PCSK9 epitope was fused to a measles virus-derived Thepitope via a flexible linker to generate the vaccine candidate. The PCSK9 peptide vaccine was synthesized by GL Ltd. (Shanghai, China), with a purity exceeding 95%, as confirmed by high-performance liquid chromatography and mass spectro­metry (MS). The purified peptides were formulated with different adjuvants and used for *in vivo* immunization studies in various animal models.

### Animals and immunization protocols

All animal studies were conducted in accordance with institutional guidelines and approved by the Animal Ethics Committee of the Changchun Institute of Biological Products. Animals were housed under standard conditions with a 12-h light/12-h dark cycle and free access to food and water. *ApoE*^−/−^ mice were purchased from Charles River Laboratories (Beijing, China) and other animals were obtained from the Changchun Institute of Biological Products Co., Ltd. (Changchun, China).

To evaluate the immunogenicity, BALB/c mice (female, 6–8 weeks old) were immunized subcutaneously with 10 μg of candidate vaccine formulated with CpG + Alum adjuvant at week 0 (prime) and week 2 (boost). Control groups received adjuvant only. Serum samples were collected at weeks 0, 2, and 4 for immunogenicity analysis.

To compare different adjuvants, BALB/c mice were immunized with 10 μg of PVC3 vaccine formulated separately with CpG, Alum, MF59, or CpG + Alum at weeks 0 and 2. Sera were collected at weeks 0, 2, and 4.

To determine the optimal antigen dose, BALB/c mice (*n *= 9 per group) were immunized subcutaneously with 2.5 μg, 10 μg, or 25 μg of PVC3 vaccine, each combined with CpG, Alum, MF59, or CpG + Alum adjuvants at weeks 0 and 2. Serum samples were collected at weeks 0, 2, and 4. In the antibody persistence study, sera were further collected every 4 weeks until week 24.

To assess the prophylactic efficacy of the PVC3 vaccine, BALB/c mice were immunized subcutaneously with 25 μg of PVC3 vaccine formulated with CpG + Alum at weeks 0 and 2. At week 2, mice were intravenously injected via the tail vein with 3 × 10^11^ vg of AAV-hPCSK9^D374Y^. Sera were collected every 2 weeks until week 14 to assess LDL-C levels and antibody titers.

To evaluate the therapeutic effect of the PVC3 vaccine, *ApoE*^−/−^ mice were immunized subcutaneously. Serum samples were collected every 2 weeks up to week 14 for evaluation of therapeutic efficacy.

Guinea pigs were immunized subcutaneously with 50 μg of PVC3 vaccine formulated with CpG + Alum at weeks 0 and 2. Serum samples were collected at weeks 0, 2, and 4 to assess antibody titers.

For NHP studies, rhesus macaques (3–5 years old, *n *= 3 per group) were immunized intramuscularly with 100 μg of PVC3 vaccine formulated with CpG + Alum at weeks 0 and 4. Blood samples were collected every 4 weeks until week 16 for immunogenicity and safety evaluation.

### PCSK9 antibody analysis

Serum anti-PCSK9 antibody titers were tested using commercialized ELISA kits according to the manufacturer’s instructions. Briefly, 96-well plates were coated with recombinant human PCSK9 protein (1 μg/mL), blocked with 3% BSA, and incubated with serially diluted serum samples. Bound antibodies were detected using species-specific HRP-conjugated secondary antibodies and 3,3',5,5'-tetramethylbenzidine (TMB) substrate. Antibody isotypes (IgG1, IgG2a, and IgG2b) were determined using isotype-specific secondary antibodies.

### Serum lipid analysis

All animals were fasted overnight prior to blood collection. Blood samples collected from the test animals were stored at 4°C overnight. Subsequently, sera were separated by centrifugation at 4000 rpm for 10 min at 4°C. Serum lipid levels, including T-CHO, TG, LDL-C, and HDL-C, were measured using biochemical assay kits (Nanjing Jiancheng Bioengineering Institute, Nanjing, China).

### Serum biochemical analysis

Serum samples were used to assess biochemical indices of kidney and liver function. Specifically, levels of BUN and CRE were measured as indicators of kidney function, while AST and ALT were evaluated as markers of liver function. All measurements were performed using commercially available assay kits (Nanjing Jiancheng Bioengineering Institute, Nanjing, China).

### Histopathology

Histological analysis was performed at the endpoint of the safety study. Mice were sacrificed and perfused transcardially with PBS, followed by 4% paraformaldehyde. After euthanasia, major organs including the heart, liver, spleen, lungs, and kidneys were collected and fixed in 4% paraformaldehyde for 24 h at 4°C. Tissues were subsequently embedded in either optimal cutting temperature (OCT) compound or paraffin, sectioned, and stained with Oil Red O or H&E.

In the efficacy study, mice were similarly sacrificed and perfused with PBS followed by 4% paraformaldehyde. Liver tissues were collected and fixed in 4% paraformaldehyde for 24 h, embedded in OCT or paraffin, sectioned, and stained with Oil Red O or H&E to evaluate hepatic lipid accumulation and general histopathology.

At the endpoint of the therapeutic assessment, the entire aorta of *ApoE*^−/−^ mice was isolated, opened longitudinally, and stained with Oil Red O to visualize atherosclerotic lesions. The lesion area was quantified as a percentage of the total aortic surface area using ImageJ software. In addition, cross-sections of the aortic root were prepared and stained with Oil Red O and H&E to assess the presence and extent of necrotic core regions.

### Elispot

Spleens were aseptically harvested from immunized mice and mechanically dissociated using the blunt end of a syringe plunger in a sterile 70-μm cell strainer. The resulting cell suspension was collected in RPMI 1640 medium supplemented with 10% fetal bovine serum (FBS). Red blood cells were lysed using ammonium-chloride-potassium lysis buffer. After washing and filtering, splenocytes were resuspended in complete RPMI and counted for downstream applications.

Peripheral blood was collected from cynomolgus monkeys in EDTA-coated tubes. The PBMCs were isolated by density gradient centrifugation using Ficoll-Paque PLUS. The mononuclear cell layer was collected, washed twice with PBS, and resuspended in RPMI 1640 supplemented with 20% FBS and 1% penicillin-streptomycin. Cells were counted and assessed for viability using trypan blue exclusion.

IFN-γ and IL-4 ELISPOT assays were performed using commercially available kits (Mabtech) according to the manufacturer’s instructions. Briefly, ELISPOT plates pre-coated with capture antibodies were seeded with 1 × 10^6^ splenocytes or PBMCs per well. Cells were stimulated in triplicate with 10 μg/mL of PEC3 or the vaccine (PVC3). Medium alone was used as a negative control. After incubation at 37°C in 5% CO_2_ for 24 h, plates were washed and incubated with biotinylated anti-IFN-γ or anti-IL-4 detection antibodies (1 μg/mL) for 2 h. This was followed by incubation with streptavidin-HRP (1:1000 dilution) for another 2 h. Spots were developed using TMB substrate for 10–20 min, washed with distilled water, and air-dried. Plates were scanned using an AID iSpot reader, and spot-forming units were quantified.

### Statistical analysis

All statistical analyses were performed using GraphPad Prism software, version 8.0 (GraphPad Software Inc., San Diego, CA, USA). Data are presented as mean ± standard deviation (SD), unless otherwise stated. Comparisons between two groups were conducted using unpaired Student’s *t*-test. For comparisons among multiple groups, one-way analysis of variance (ANOVA) followed by Tukey’s *post hoc* test was used. For time-course, two-way ANOVA followed by Sidak’s *post hoc* test was applied. A *P* value of less than 0.05 was considered statistically significant. Statistical significance is indicated in the figures as follows: *P < *0.05 (*), *P* < 0.01 (**), *P* < 0.001 (***), and ns denotes no significant difference.

## Supplementary Material

loag013_Supplementary_Data

## Supplementary data

**Author contributions** 

Conceptualization and supervision: Ruiping Xiao; Study sesign and resources: Hongliang Sun; Writing - original draft and software: Zhuang Li; Investigation, data curation, and methodology: Kun Ma, Jian Zhang, and Chang Liu; Writing - review & editing: Xuemei Zhang and Xinli Hu.

R.X. holds the position of Editorial Board Member for *Life Metabolism* and was blinded from reviewing or making decisions for the manuscript. The other authors declare that no conflict of interest exists.

**Ethics approval ** 

All animal studies were conducted in accordance with institu­tional guidelines and approved by the Animal Ethics Committee of the Changchun Institute of Biological Products.

**Data availability ** 

All the data supporting the findings of this study are available within the supplementary material and corresponding authors.
