# Supplementary material for: Structure-guided design of a PCSK9 epitope vaccine with efficacy against hyperlipidemia and atherosclerosis
Source: Life Metab. 2026 May 26;5(4):loag013. doi: 10.1093/lifemeta/loag013 (PMC13322968; doi:10.1093/lifemeta/loag013)
Supplement: loag013_Supplementary_Data [file loag013_supplementary_data.docx]

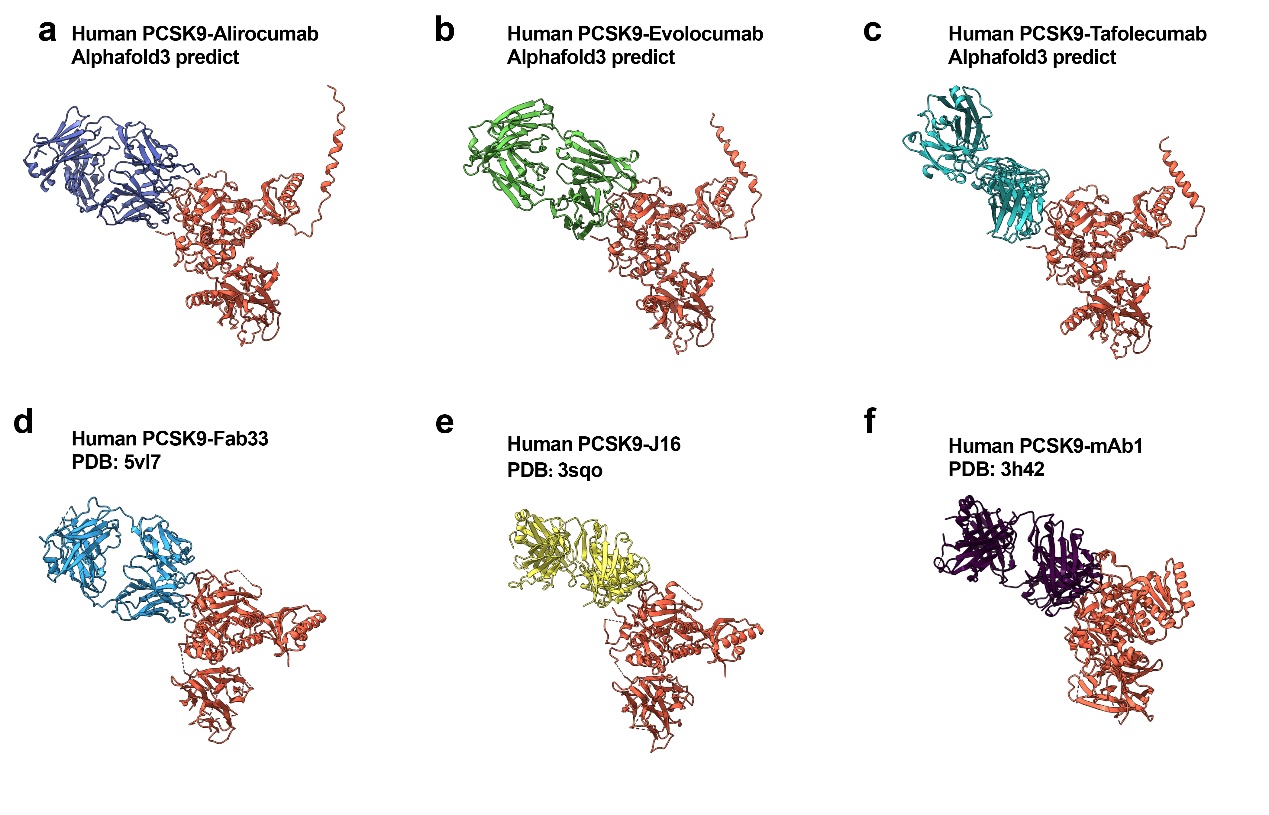


## Supplementary Figure S1 The structure of PCSK9 and the antibody. (a−c) The structure of PCSK9 and antibody complex predicted by Alphafold3. (d−f）The structure of PCSK9 and the antibody retrieved from the PDB repository.

##
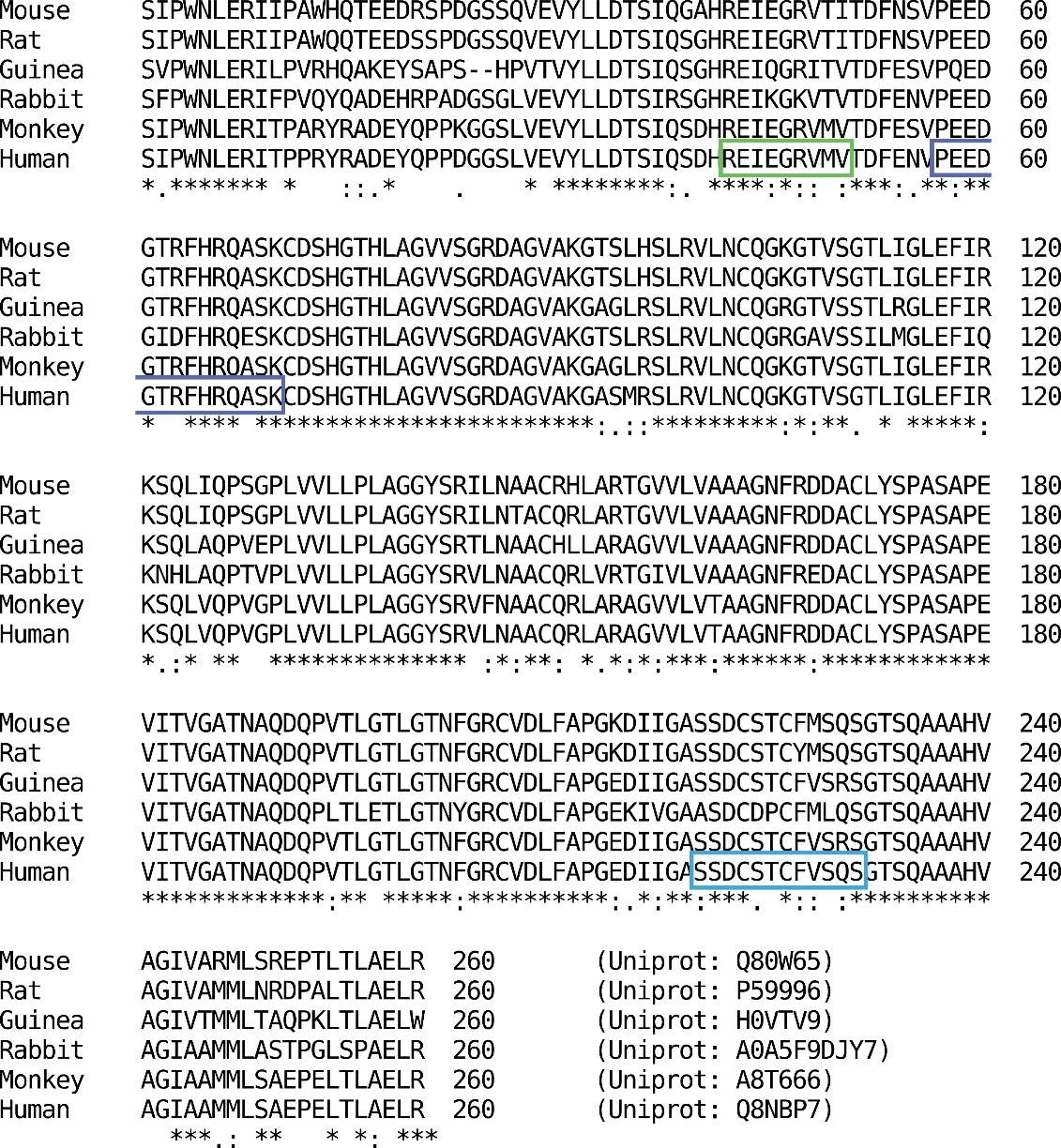
 Supplementary Figure S2 PCSK9 sequence alignment from different species.
